# Enhancing Clay-Based 3D-Printed Mortars with Polymeric Mesh Reinforcement Techniques

**DOI:** 10.3390/polym16152182

**Published:** 2024-07-31

**Authors:** Sotirios Pemas, Konstantina Sougioultzi, Chrysoula Kouroutzidou, Maria Stefanidou, Avraam A. Konstantinidis, Eleftheria Maria Pechlivani

**Affiliations:** 1 Centre for Research and Technology Hellas, Information Technologies Institute, 6th km Charilaou-Thermi Road, 57001 Thessaloniki, Greece; sopemas@iti.gr; 2 Laboratory of Building Materials, School of Civil Engineering, Aristotle University of Thessaloniki, 54124 Thessaloniki, Greece; ksougi@civil.auth.gr (K.S.); ckourout@civil.auth.gr (C.K.); stefan@civil.auth.gr (M.S.); 3 Laboratory of Engineering Mechanics, School of Civil Engineering, Aristotle University of Thessaloniki, 54124 Thessaloniki, Greece; akonsta@civil.auth.gr

**Keywords:** additive manufacturing (AM), 3D printing, liquid deposition modeling (LDM), fused filament fabrication (FFF), clay-based mortars, 3D-printed mortars, 3D-printed reinforcement, mechanical properties, innovative mortars, sustainable construction

## Abstract

Additive manufacturing (AM) technologies, including 3D mortar printing (3DMP), 3D concrete printing (3DCP), and Liquid Deposition Modeling (LDM), offer significant advantages in construction. They reduce project time, costs, and resource requirements while enabling free design possibilities and automating construction processes, thereby reducing workplace accidents. However, AM faces challenges in achieving superior mechanical performance compared to traditional methods due to poor interlayer bonding and material anisotropies. This study aims to enhance structural properties in AM constructions by embedding 3D-printed polymeric meshes in clay-based mortars. Clay-based materials are chosen for their environmental benefits. The study uses meshes with optimal geometry from the literature, printed with three widely used polymeric materials in 3D printing applications (PLA, ABS, and PETG). To reinforce the mechanical properties of the printed specimens, the meshes were strategically placed in the interlayer direction during the 3D printing process. The results show that the 3D-printed specimens with meshes have improved flexural strength, validating the successful integration of these reinforcements.

## 1. Introduction

Over time, ancient monuments and buildings from various chronological periods undergo deterioration primarily caused by environmental factors, weather conditions, and lack of maintenance knowledge [1,2]. These historical buildings face challenges such as air pollution, salts, biodeterioration, acid rain, and earthquakes [1,3,4]. Among historic structures, those of vernacular architecture occupy a significant part [5]. It is crucial to find ways to maintain and restore these structures to preserve ancestral heritage and evidence of past civilizations, as they provide cities with a sense of identity and community. Therefore, preserving or repurposing these structures is essential, as history holds valuable lessons for us [6].

Although restoration processes often involve meticulous historical research and the use of traditional techniques and original materials, there are circumstances where traditional methods are impractical or original materials are unavailable [2,7]. Furthermore, there are obstacles to restoration tasks, such as reproducing missing parts due to the lack of drawings and documentation, restrictions on preservation tools, restrictions on direct touch, and a shortage of skilled artisans capable of replicating complicated details [1,8,9]. Fortunately, modern technologies offer automatic restoration methods to aid conservation processes [10,11].

To overcome these challenges, advancements in digital technology, such as three-dimensional (3D) scanning and photogrammetric shape measurement systems, allow for non-contact and non-destructive measurements of existing structures and monuments [12,13,14,15]. A perfect synergy is achieved with 3D printing technologies, which offer a wide variety of technologies and materials for restoring historical buildings, monuments, sculptures, and ornaments [16,17,18,19]. These technologies can be employed in various applications, such as preserving heritage buildings. For instance, they can 3D print supportive structures and joints for masonry infills [20,21,22]. Liquid Deposition Modeling (LDM), in particular, can use a diverse range of building materials as feedstock [23,24,25]. For restoration purposes, building mixtures that comply with regulations and harmonize with the original material in terms of color, texture, and physical and mechanical properties can be utilized [1,26,27,28]. Furthermore, it is worth noting that recent studies have demonstrated 3D printing processes capable of manufacturing parts with rock-like materials [29,30,31]. Such advancements can produce final parts with more realistic appearances, which is beneficial for applications such as restorations.

Especially for building and construction purposes, many commercial and customized 3D printing systems have been developed [32,33], such as additive manufacturing (AM) of concrete known as 3D concrete printing (3DCP) and LDM technology, garnering attention from academia and industry [34]. Currently, 3D printing companies for building materials, such as WASP, which has developed a Crane system for rapid and sustainable 3D-printed house construction, are showcasing the capabilities of 3D printing technology and earthen-based architecture through project TECLA [35]. Among the numerous customized solutions found in the literature for 3DCP, Delft University of Technology designed a lab-scale 3DCP setup comprising a computer numerical control (CNC) machine, controller, and extrusion system, with the CNC operated by the controller and material directed to the nozzle via a commercial PFT Swing-M conveying pump [34,36]. Another system developed by Manikandan et al. [37] involved an extrusion-based Velleman K8200 3D printer with a 20 cm × 20 cm × 20 cm build volume and a 3 mm diameter nozzle for printing and characterizing ready-to-print fresh cementitious mixtures with silica fume and superplasticizer. In addition, robotic arms offer an attractive solution for 3D printing concrete mixtures due to their freedom of movement, allowing for various applications, from small objects to larger constructions. They can also be used for printing simultaneously in collaboration with other robotic arms, contributing to the elimination of building time for the project [38,39]. Alabbasi et al. [40] employed robotic 3D printing tools and techniques for building typical Saudi Arabian free-standing houses, while Xu et al. [41] introduced a mobile robotic 3D concrete printing system for in situ construction, utilizing robotic arms for printing various structural components of a farmhouse in a village, including foundation trenches, structural walls, decorative walls, arch roofs, and flat roofs.

Generally, according to Cao et al. [42], the 3DCP systems found in the literature can be categorized as Gantry, Frame, Robot arm, Polar, and Delta types [39,42]. Gantry and frame systems are commonly adopted in the Fused Filament Fabrication (FFF) technology and can be easily converted for concrete printing. They are known for their low cost, ease of assembly, and maintenance. Robot arm systems offer flexibility and the ability to print complex geometries, with the potential for extendable build volume if the base can be moved. The drawback of those systems is that they come with high costs and complexity in control. Polar systems, less common in academic settings, are efficient for commercial projects like house printing, featuring a cylindrical build volume but with higher maintenance and fabrication costs. Delta systems, very common in FFF printers, can be found in small or large sizes and can be easily converted for concrete printing, but they face stability issues due to their structure.

This study presents a rapid methodology for restoring monuments and buildings, adhering to specific material legislation. The goal is to inspire civil engineers and architects to leverage additive manufacturing technologies in restoration projects to take advantage of the numerous benefits it offers. Additionally, it explores enhancing the mechanical properties of clay-based printed parts for restoration by incorporating polymer meshes. In detail, four different clay-based mixtures were developed: 1. 100% Clay, 2. 95% Clay—5% Cement, 3. 90% Clay—10% Lime, and 4. 80% Clay—20% Lime. These mixtures were printed using LDM technology, both with and without integrated polymer meshes made of PLA, ABS, and PETG for extra reinforcement, and then evaluated to determine the best option and the most favorable mechanical properties. The materials proposed were based on lab experience and the most usual ways to stabilize clay [43]. Thus, in addition to the aforementioned per-weight percentages, the water content was adjusted in order to provide the best properties to the various mortars, ceramic powder was used as an aggregate, and a plasticizer was also used to reduce water content, as mentioned in the next section. It was considered safe to use white cement at a percentage of 10% of binders for repair mortars, as physical properties (such as porosity and capillarity) are not significantly affected. Additionally, the early strength is increased to support the materials and the construction [44,45].

**Figure 1:**
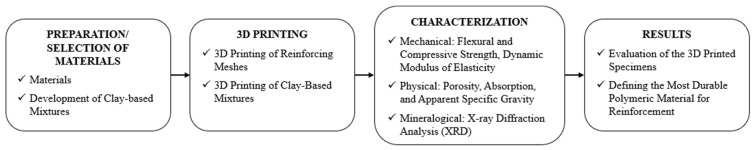
Flowchart of the present study.

Figure 1 illustrates the flowchart of the present study and essentially the methodology followed for developing clay-based mixtures for 3D printing applications and their assessment. Section 2 of this study presents the materials and methods, explaining the methodology and equipment used to develop and assess clay-based mixtures and utilize them through LDM 3D printing. Section 3 showcases the results concerning the chemical composition of the mixtures, characterization results of the printed prisms, and the resulting mechanical properties. The study concludes with Section 4, which provides a brief summary of the methodology and results.

## 2. Materials and Methods

### 2.1. Materials

**Figure 2:**
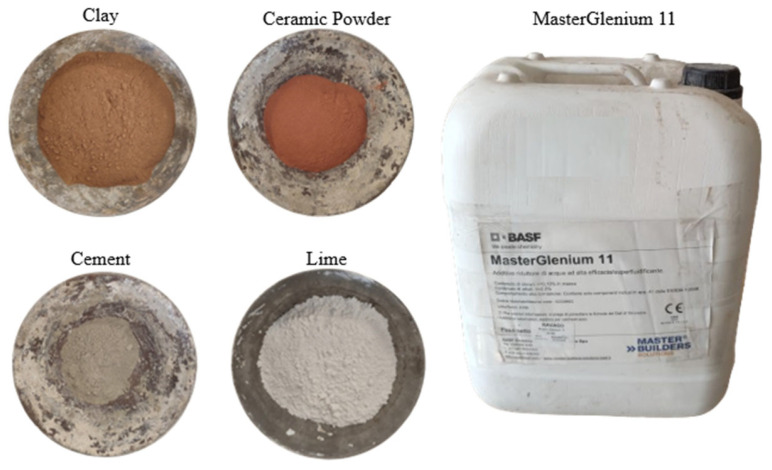
Materials utilized in the formulation of clay-based mixtures.

For this study, clay-based mortars were prepared in the laboratory in two different groups for comparison. One group of specimens was printed, and the other was molded. Table 1 summarizes the mortar series made—the raw materials used as binders were clay, hydrated lime, and cement I42.5N in different proportions. Ceramic powder, derived from crushing bricks available at the market, was used as an aggregate. The hydrated lime and cement were intended as stabilizers. Stabilizers are usually hydraulic (primary binders) or non-hydraulic (secondary binders) materials that react with water primarily or in the presence of pozzolanic materials (secondarily) to form composites with adhesive properties. In an attempt to decrease water usage, a polycarboxylate superplasticizer (Master Glenium 11) supplied by BASF (Ludwigshafen, Germany) was incorporated in different proportions into each mixture. It is a new generation reducing admixture based on modified polycarboxylic ether chains, mainly developed for the ready-mix concrete industry. Figure 2 illustrates the materials investigated in the present study.

The analysis of the raw materials was conducted using X-ray fluorescence (XRF) for chemical analysis and a gas pycnometer for density measurement. Additionally, the particle size of the binders was measured. For XRF analysis, a Bruker TIGER S8 (Karlsruhe, Germany) was used. Density was measured using Ultrapyc 3000 from Anton Paar (Graz, Austria), and particle size was measured with a Mastersizer 2000 from Malvern Panalytical (Malvern, UK). The properties of the binders are summarized in Table 1.

### 2.2. Preparation Process of Clay-Based Mortar Mixtures

**Figure 3:**
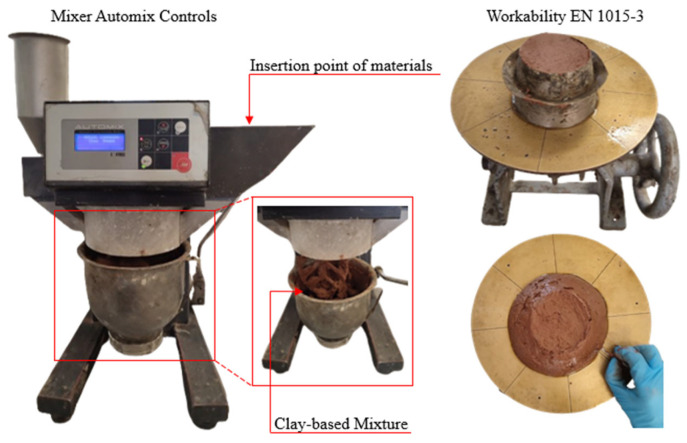
Preparation of clay-based mixtures.

In this research study, the clay-based mixtures were prepared using an automatic programmable mixer, specifically the CONTROLS 65-L0006/AM (Liscate, Milan, Italy), featuring a planetary rotation system for its working body (refer to Figure 3).

The mortar samples were produced according to EN1015-11 [46]. The specimens were prisms with dimensions 4 cm × 4 cm × 16 cm. Water and Master Glenium 11 were first poured into the mixer bowl, followed by the addition of clay. Then, the cement or lime was added to the respective clay-based mixture. Subsequently, the mixer was turned on at low speed and mixed the materials for 30 s. Following this stage of mixing, the ceramic powder was added, and the mixing continued at a low speed. Finally, the mixer was switched to high speed for 60 s. The mortar series created are summarized in Table 2.

To assess the workability of the fresh-state mortar according to EN 1015-3 [47], flow table consistency was measured. The conical mold was lubricated, filled with the mortar mixture, and compacted to remove air bubbles. After removing the mold, the slump flow table was dropped from the standard height at a constant rate for fifteen consecutive times. This resulted in a final diameter increase of 14 cm. This method not only determines flowability but also provides insight into the shape retention ability and, by extension, the extrudability of the fresh 3D-printable mixture.

### 2.3. Design and 3D Printing of Reinforcing Meshes

To propose a methodology for achieving superior mechanical performance in constructions made by 3D printing (with layer-by-layer deposition of the building material) and to overcome weaknesses such as poor interlayer bonding and material anisotropies, reinforcing meshes with modified surfaces for better adhesion between the layers were designed and 3D printed.

This aims to achieve the optimal structural strength of the clay-based printed specimens. From the literature, a highly durable design known as the Quadri-Grid structure was chosen for this research due to its demonstrated performance in bending capacity and mechanical properties in three-point bending tests [48]. The meshes were fabricated using Fused Filament Fabrication technology (FFF). All specimens were designed using SOLIDWORKS^®^ CAD Software (2022 SP2.0 Professional version) and were 3D printed using an Original Prusa i3 MK3S+ 3D printer (Prusa Research a.s., Prague, Czech Republic). Additionally, Prusa Slicer 2.5.0 software was utilized to slice the geometry and export the G-code. It is noteworthy that, in this study, the Quadri-Grid structure was modified by adding scattered circular speckles to enhance adhesion between layers. The dimensions of the mesh are 151 mm × 31 mm × 1 mm (length × width × height), with the scattered speckles positioned 0.5 mm from the mesh surface. Consequently, the total height of the mesh is 1.5 mm, resulting in overall dimensions of 151 mm × 31 mm × 1.5 mm (Length × Width × Height).

**Figure 4:**
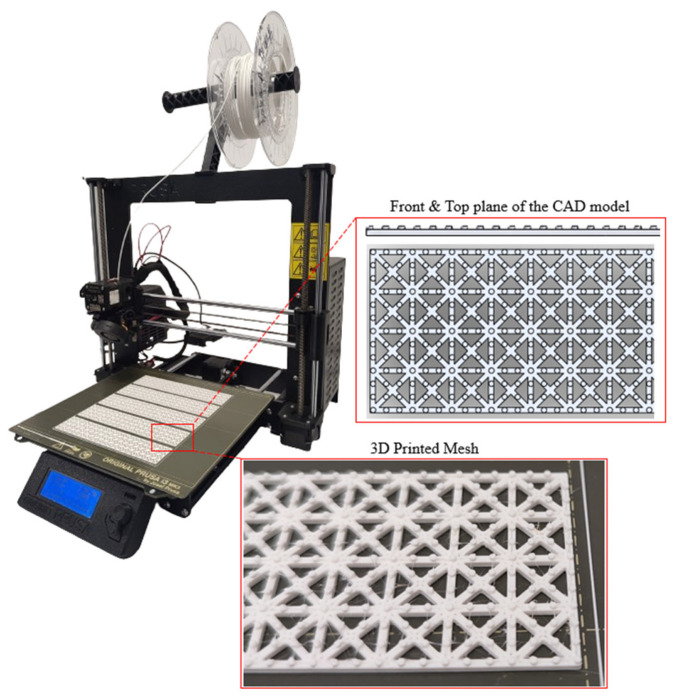
Three-dimensional printing of reinforcing meshes.

To determine the most suitable material in terms of mechanical properties and adhesion between layers of the clay-based mortar, three widely used materials in FFF technologies were selected: PLA, PETG, and ABS. All materials were provided in filament form with a diameter of 1.75 mm. More specifically, the filaments used are eSUN PLA+ (Shenzhen, China), eSUN PETG (Shenzhen, China), and Spectrum Smart ABS (Pęcice, Poland). The printing parameters were defined, with the nozzle temperature set at 215 °C for PLA, 240 °C for PETG, and 255 °C for ABS. Bed temperatures were set at 60 °C for PLA, 90 °C for PETG, and 110 °C for ABS. All meshes were printed using a 0.4 mm nozzle and 0.2 mm layer height, with a fill density of 100% using a rectilinear fill pattern. Figure 4 demonstrates the FFF 3D printer utilized, a section of the CAD design of the mesh, and illustrates the printed meshes on the printing bed.

### 2.4. Three-Dimensional Printing of Clay-Based Mortar Mixtures

The additive manufacturing technology employed in this study for the fabrication of clay-based specimens is Liquid Deposition Modeling (LDM). All specimens were designed using SOLIDWORKS^®^ CAD Software (2022 SP2.0 Professional version) and were 3D printed using the WASP 40100 LDM. Simplify3D (Version 5.1.2) software was utilized to define the printing parameters and export the G-code.

The fresh mixture was produced according to EN1015-11, and specimens were designed with dimensions (4 cm × 4 cm × 16 cm) to mimic the molded samples. Subsequently, the pump of the printer was filled with the fresh clay-based mixture and prepped to ensure the least amount of encapsulated air and voids.

Regarding the printing process, the employed nozzle diameter was 10 mm, and the layer height was set at 5 mm for the printing parameters. Finally, the fill density was set at 100%. All mixtures were prepared and printed at room temperature.

After uploading the G-code to the printer, the mixture was imported into the container of feedstock material, and with the help of a piston inside this container, the mixture was pushed through a pipe into the extruder. The extruder has a cylindrical shape and contains a screw that pushes the mixture toward the nozzle. The rotational speed of the screw and the pressure exerted on the piston determine the flow rate of the extruded material. The pressure exerted on the piston was set at 0.1 MPa, and the compressed air that pushes the piston is generated from an air compressor connected to the top part of the container containing the mixture.

For the present study, a total of eighteen prisms were printed. Four different mixtures were developed (exactly the same as the molded ones—see Table 2), and three prisms were printed with each mixture. Additionally, six prisms were printed with integrated polymeric mesh reinforcements: three with the clay mixture and three with the clay and 5% cement mixture. As already mentioned, the meshes were positioned between all layers. Specifically, for each prism consisting of eight layers, seven polymeric meshes were placed. It is noted that although only the 3D-printed meshes below the neutral axis would undertake tensile loads, it was decided to place them between all layers because it is expected to enhance the layers’ cohesion as well [49,50]. The 3D printing process was stopped after completing every layer, and the mesh was positioned manually. Table 3 presents all the printed prisms analytically.

**Figure 5:**
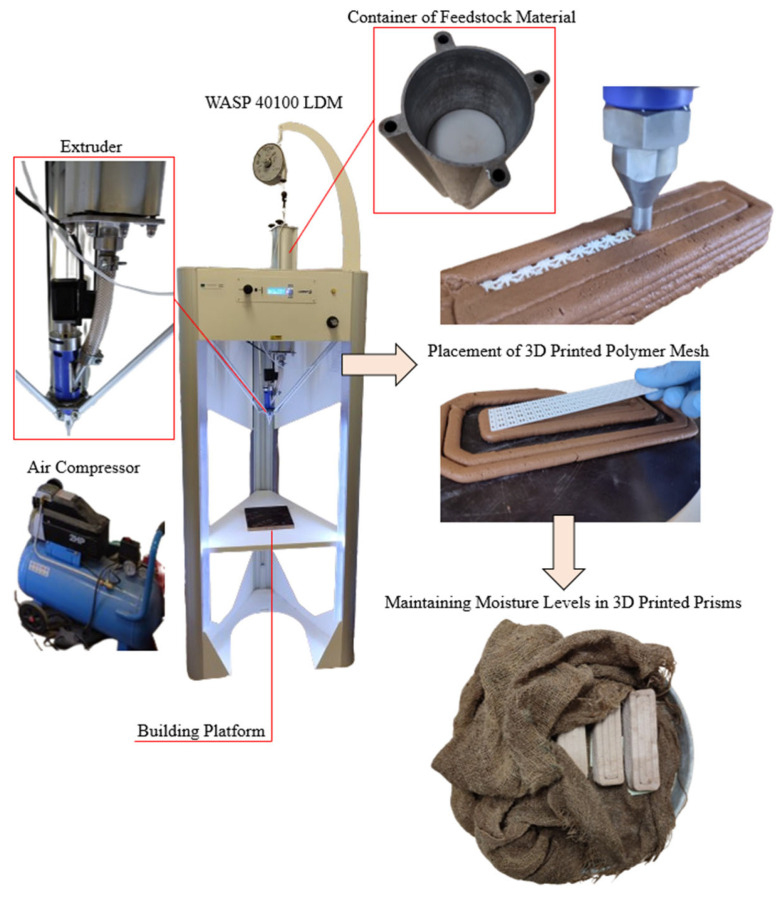
Three-dimensional printing of clay-based prisms.

After the printing process, the specimens were maintained in a humid environment. To achieve a humid environment and prevent cracking, the specimens were wrapped in wet burlap after three days and stored at room temperature (20 ± 2 °C) with 95% relative humidity. Figure 5 displays the WASP LDM 3D printer, the printing process, and the maintenance of the printed prisms.

### 2.5. Preparation of Molded Specimens

**Figure 6:**
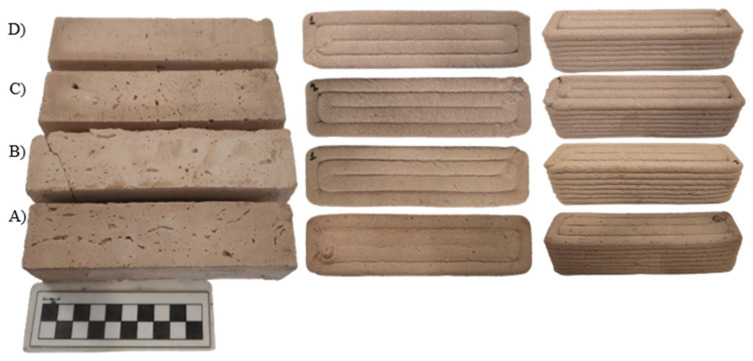
Final casted and 3D-printed prisms. (**A**) 100% Clay, (**B**) 95% Clay—5% Cement, (**C**) 90% Clay—10% Lime, (**D**) 80% Clay—20% Lime.

Prismatic metallic molds (4 cm × 4 cm × 16 cm) were lubricated and filled with the fresh-state mixture to prepare the molded specimens. Tamping on a vibration table for 30 s removed air bubbles and voids. After 3 days, the de-molded specimens were cured under the same conditions as the printed ones: in wet burlap at room temperature (20 ± 2 °C) and 95% relative humidity. The final cast and 3D-printed prisms are shown in Figure 6.

### 2.6. Materials Characterization

The mechanical, physical, and mineralogical properties of the prismatic specimens were evaluated after curing. Regular molded and 3D-printed specimens were cured for 90 days, while specimens with reinforcing meshes were cured for 28 days. The curing process involved humid conditions of 95% relative humidity and a temperature of (20 ± 2) °C for the first 28 days, followed by storage in a laboratory environment.

#### 2.6.1. X-ray Diffraction (XRD)

X-ray diffraction analysis (XRD) was employed to evaluate the phase composition of the four mortars produced in this study. A Bruker 2nd generation D2-Phaser diffractometer was used for the XRD analysis. X-ray diffraction patterns were recorded at Cu Ka (30 kV and 10 mA, λ  =  1.540 Å) from 2° θ to 75° θ, with step 0.02° θ and time per step 0.4 s. The crystalline phases were identified using DIFFRAC.EVA V5.0 software and the Crystallography Open Database.

#### 2.6.2. Physical Properties

The porosity, absorption, and apparent specific gravity of the clay-based specimens were determined according to the RILEM CPC 11.3 method, which utilizes water absorption under vacuum.

#### 2.6.3. Mechanical Properties

The flexural and compressive strength was determined using a model WAW-300E computer-controlled Universal Testing Machine, according to EN 1015-11:2019 [46]. Mechanical properties were tested at three months (90 days) [43,51,52] on the molded and unreinforced 3D-printed samples and at 28 days on the 3D-printed specimens with reinforcing meshes.

To account for the anisotropic nature of 3D-printed specimens, compressive testing was performed in two directions: parallel and perpendicular to the printed layers. Flexural strength, however, was only measured perpendicular to the layers. In the case of 3D-printed specimens with reinforcing meshes, only flexural strength was assessed due to their geometry causing delamination during the test.

To assess the dynamic modulus of elasticity according to EN 12504-4 [53], ultrasonic propagation, a non-destructive technique, was employed. This method determines the velocity of elastic waves, allowing for the detection of voids and discontinuities within the material. A Proceq PUNDIT, a portable ultrasonic non-destructive digital indicating tester equipped with two 54 kHz cylindrical P-wave transducers, was used for this purpose.

## 3. Results

### 3.1. X-ray Diffraction (XRD) Measurements

**Figure 7:**
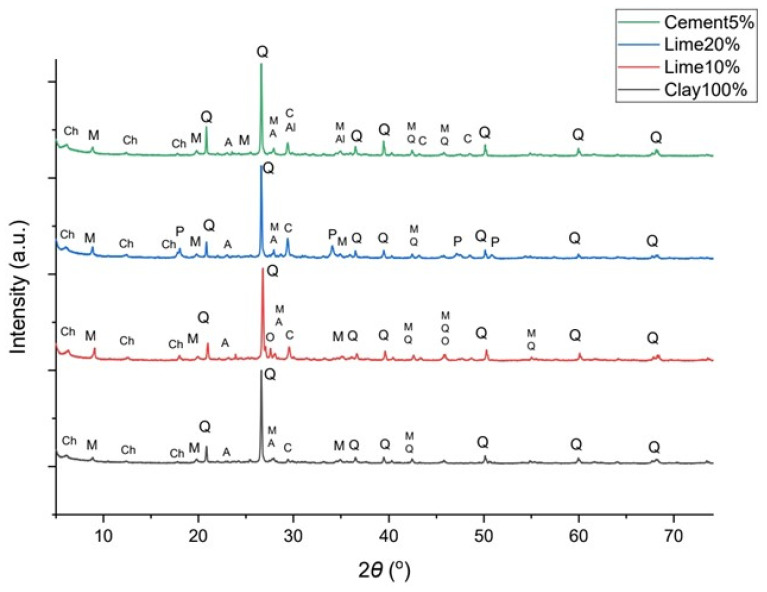
XRD patterns of the 100% clay specimen, 10% lime, 20% lime and 5% cement mortars. Q: quartz, C: calcite, M: muscovite, A: albite, Al: alite, Ch: chlorite, P: portlandite.

The crystalline phases identified in the clay-based samples after 28 days are presented in Figure 7.

The X-ray diffraction (XRD) pattern of the clay mortar shows the identified crystalline phases, including quartz (COD 1011159), muscovite (COD 9001952), albite (COD 9009663), chlorite (COD 9000158), and calcite (COD 1010928), all present in very low concentrations. The XRD pattern of the mortar containing 10% lime reveals the presence of quartz (COD 1011172), muscovite (COD 9001952), albite (COD 9002196), chlorite (COD 9000158), calcite (COD 9007689), orthoclase (COD 1011205), and portlandite (COD 1001768). The mortar with 20% lime exhibits similar phases. However, the calcite peak shows a significant increase compared to the 100% clay mortar. This can be attributed to the addition of hydrated lime, while the presence of portlandite peaks suggests the occurrence of hydration. Based on the analysis of the XRD patterns, no clear evidence of a pozzolanic reaction between the clay minerals and the hydrated lime is observed.

The XRD pattern of the 5% cement mortar reveals the presence of quartz (COD 1011097), muscovite (COD 9001952), calcite (COD 9000965), albite (COD 9002197), orthoclase (COD 9000161), chlorite (COD 9000158), and alite (COD 9016125). Alite, the primary component of Portland cement, undergoes hydration to form calcium silicate hydrate (C-S-H) and calcium hydroxide (portlandite), which are crucial for cement setting and strength development of the matrix [54,55,56]. While alite was present, no hydration products were found. 

While lime and cement were used to stabilize the clay and promote the formation of pozzolanic compounds such as calcium silicate hydrate and calcium aluminate hydrate, these phases were not detected by XRD analysis. Pozzolanic compounds play a role in reducing swelling and increasing strength in clay. The absence of these pozzolanic phases suggests a lack of pozzolanic activity, which may compromise the long-term mechanical performance and durability of the clay-based mortars. 

### 3.2. Physical Properties

**Figure 8:**
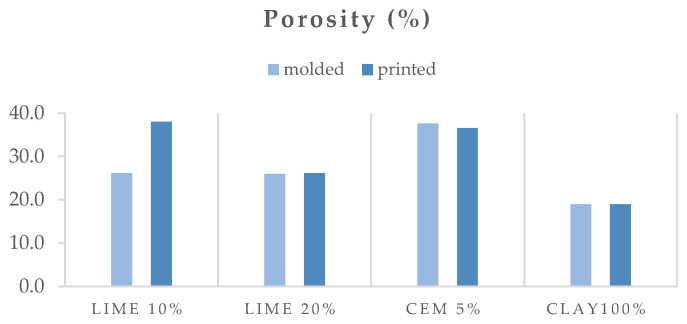
Open porosity (%) of the molded and printed specimens.

**Figure 9:**
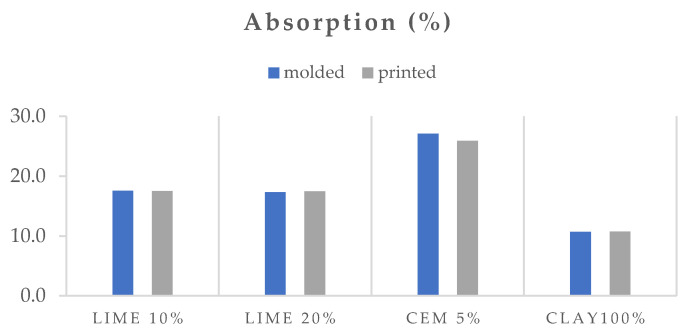
Absorption (%) of the molded and printed specimens.

**Figure 10:**
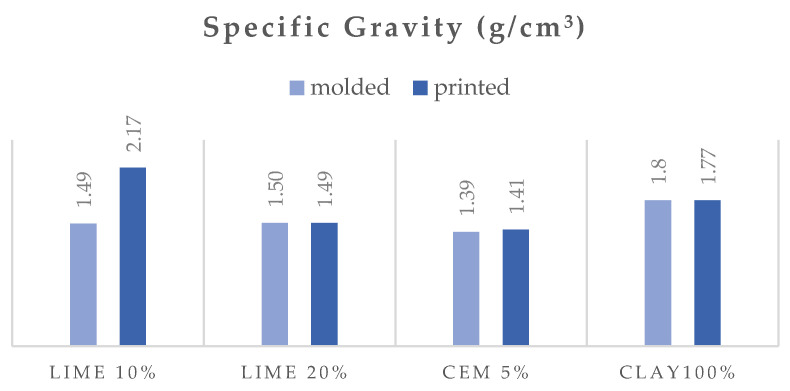
Specific gravity of the molded and printed specimens.

Figure 8, Figure 9 and Figure 10 show the open porosity (%), absorption (%), and specific gravity of the molded and printed specimens. In most cases, the open porosity appears similar between molded and printed specimens, with the exception of the lime 10% specimen, which exhibits a significant increase in porosity for the 3D-printed version. This can be attributed to the preparation methods. The molded lime 10% specimens were compacted using a vibration table according to EN 1015-11, which reduces air pockets and ensures a denser material. In contrast, the 3D-printed specimens did not undergo external compaction, resulting in higher porosity in the lime 10% due to trapped air during the printing process. The clay and cement specimens inherently settle and compact sufficiently during both molding and 3D printing. The specific gravity follows a similar trend, with lime at 20%, cement at 5%, and clay at 100%, showing minimal differences between molded and printed specimens. However, the printed lime 10% mortar has a noticeably higher specific gravity compared to its molded counterpart. Interestingly, the absorption remains consistent across all molded and printed specimens within each mortar series.

The mortar with 100% clay has lower porosity and absorption than the mortars with hydrated lime and cement. This could be due to the smaller amount of water added to the mixture to achieve workability, as well as the particle size distribution and clay’s ability to pack densely. It is evident that the addition of cement increases porosity and absorption the most compared to mortars with other binders. This may be because cement does not have a plethora of cementitious phases that occupy space in the microstructure of the matrix.

### 3.3. Mechanical Properties

**Figure 11:**
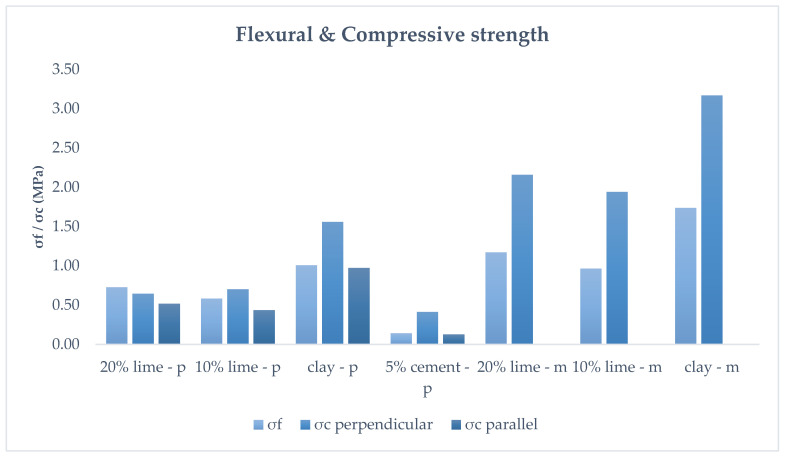
Flexural and compressive strength of the molded (m) and printed (p) specimens at 3 months.

The mechanical properties of the mortar series tested after 3 months of curing are exhibited in Figure 11.

**Figure 12:**
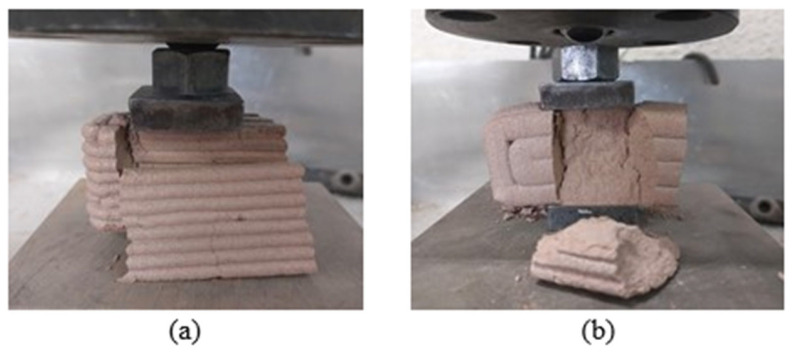
Compressive strength: (**a**) perpendicular and (**b**) parallel to the printed layers.

Due to the anisotropic properties of the 3D-printed mortars, the compressive strength was measured in both parallel and perpendicular directions, as illustrated in Figure 12.

Contrary to expectations, the mortars containing stabilizers, namely lime and cement, exhibited lower mechanical performance than the pure clay mortar. On the other hand, it was expected that the molded specimens would have superior mechanical properties compared to the printed specimens due to their printing orientation. Since the material is deposited in layers, the strength of the bond between the layers is weaker than the strength within a uniform layer. As can be seen in Figure 12, during testing, the specimens failed by layers detaching from each other.

Both molded and 3D-printed specimens of pure clay demonstrated the highest flexural and compressive strengths. The addition of 5% cement resulted in an adverse effect. The molded specimens failed during the curing process, hindering a proper strength evaluation. The printed specimens underperformed, exhibiting 86% lower flexural strength and 74% lower perpendicular compressive strength compared to the printed clay mortars.

The effect of lime inclusion provided mixed results. Although it did not perform better than the clay mortars, it showed a more hopeful outcome. By examining the two percentages used, the 20% lime mortar displayed improved flexural strength compared to the 10% lime mortar. However, its compressive strength was less than the 10% lime mortar in the perpendicular to the printed layers direction. Conversely, the 20% lime mortar maintained better compressive strength in the direction parallel to the layers. The molded lime mortars showed a clearer trend: a higher percentage of lime resulted in enhanced mechanical properties.

The results reveal that the stabilization of the clay was not successful. This could be due to several factors. Most importantly, the water content might not have been optimal for the complete hydration of the cement, especially when using a high percentage of plasticizer. Incomplete hydration of the cement limits the formation of strong binding phases that promote enhanced mechanical properties. Additionally, the curing conditions could have hindered the stabilization of the clay–cement mortar.

**Figure 13:**
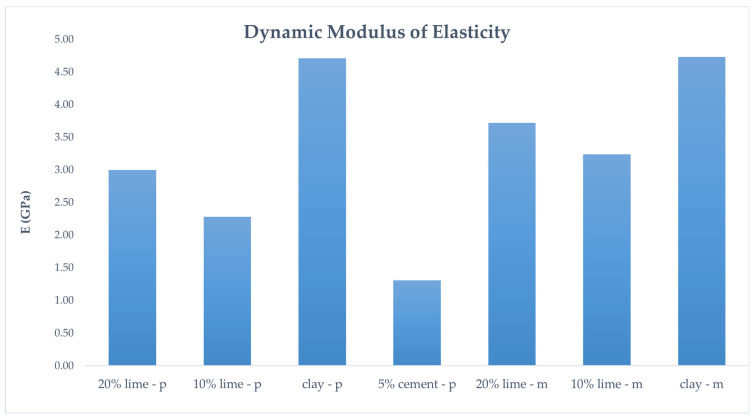
Dynamic modulus of elasticity of the molded and printed specimens at 3 months.

In Figure 13, the dynamic moduli of elasticity of the molded and printed specimens are depicted. Both printed and molded clay specimens show the same dynamic modulus, while the lime mortars show the same pattern, with the 20% lime mortar having a greater elastic modulus than the 10% lime mortar. The significantly lower dynamic modulus in the 5% cement mortar could be due to incomplete cement hydration. As discussed earlier, insufficient water might have hindered complete hydration, leading to a more porous microstructure (seen in Figure 8). In addition, there could be an incompatibility between the specific clay and cement, resulting in weak interfacial bonds and possibly some internal voids.

**Figure 14:**
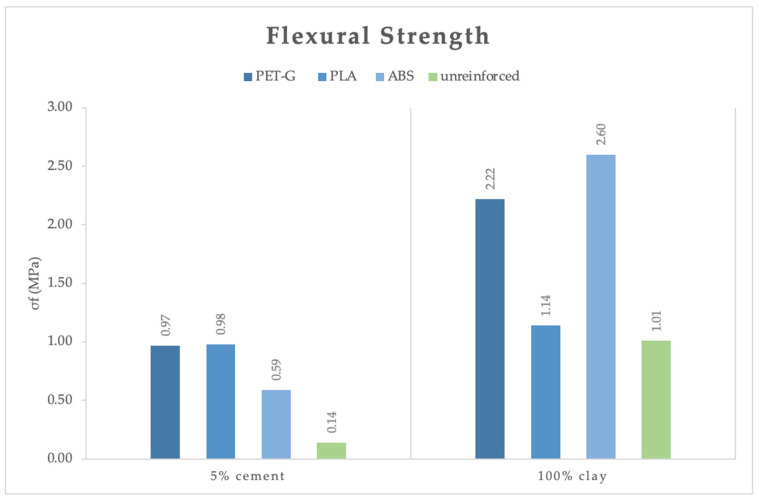
Flexural strength of the specimens printed with reinforced meshes at 28 days.

The mechanical properties of the mortar series with integrated meshes, tested after 28 days of curing, are exhibited in Figure 14. PETG, PLA, and ABS meshes were placed between printing layers in two different mortars: 100% clay and 5% cement. Firstly, it can be seen that the meshes, even after 28 days, have increased flexural strength. Again, it is clear that the 100% clay mortar provides higher flexural strength than the 5% cement mortar.

**Figure 15:**
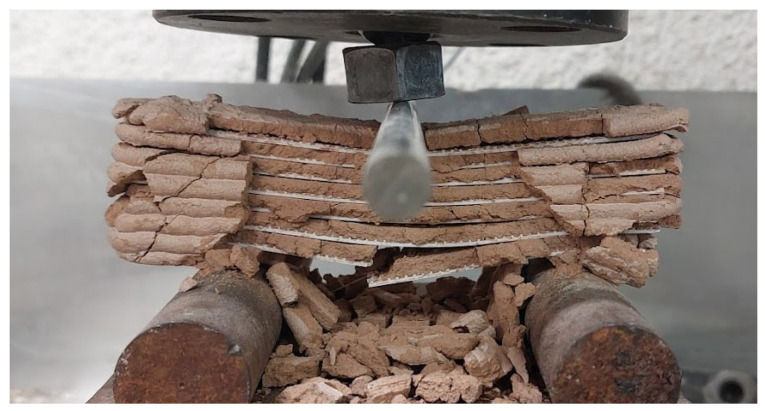
Flexural strength of 3D-printed specimens with reinforcing meshes tested perpendicularly to the printed layers.

During the test, shear failure was observed in all mortars, with two diagonal cracks propagating down the specimen from the loading point. The layers then began to detach from each other one by one until the meshes, in most cases, started breaking in half. The stiffness of the polymeric meshes was an important factor, along with their integration with the matrix. In the 5% cement mortar, PETG meshes did not break because the poor integration of the two materials, combined with the flexibility of the mesh, caused the printed layers to separate quickly, leading to composite failure. This issue was avoided in the 100% clay mortar due to better incorporation of the materials. The PLA and ABS meshes failed with a central crack starting from the bottom mesh, and most of the time, one or two broke. Figure 15 demonstrates the failure of a 5% cement specimen reinforced with an ABS mesh.

In comparison to the 3D-printed mortars without meshes, the PETG meshes increased flexural strength by 593% in the 5% cement and 120% in clay mortars, respectively. PLA meshes resulted in a 600% increase in the 5% cement mortar but only a 13% increase in the clay mortar. This poor enhancement is possibly due to the lower mechanical properties of PLA compared to PETG and ABS, as well as the poor adhesion of the PLA mesh to the clay mortar. ABS meshes strengthened the 5% cement and clay mortars by 321% and 157%, respectively. Very importantly, the addition of PETG and ABS meshes assisted the 3D-printed pure clay mortars in surpassing the flexural strength of the molded one by 28% and 49%, respectively.

One observation made before the test was that the clay mortar had quite a few superficial cracks, while the cement mortars did not. The main cause is that the meshes constrained the clay mortar from shrinking, especially in breadth. Despite the presence of superficial cracks, the clay mortar seemed to have integrated better with the meshes, resulting in better mechanical properties and enhanced cohesion of the printed layers.

## 4. Conclusions

The present study demonstrates an approach utilizing AM technologies that can be employed for restoration projects. Four different mixtures, compliant with regulations regarding allowable materials and proportions, were developed and investigated for their 3D printing potential. Their printability was successfully confirmed, and prisms with dimensions of 4 cm × 4 cm × 16 cm were produced according to EN1015-11. These prisms were designed and printed for further evaluation of their mechanical properties. Additionally, to further enhance their mechanical strength, polymer meshes made of PLA, ABS, and PETG were added between the layers of selected mixtures (100% clay and 5% cement) as extra reinforcement.

The results regarding their mineralogical analysis and physical and mechanical properties indicate that mix designing and the optimal concentrations are of paramount importance for both printed and molded clay-based materials, as selected binders, additives, and water content significantly affect the long-term properties. The XRD analysis revealed the presence of key crystalline phases, including quartz, muscovite, albite, chlorite, and calcite, in varying concentrations across different mortar compositions. Notably, the addition of lime increased the calcite content and introduced portlandite, yet no clear evidence of pozzolanic reactions was observed in any of the mortars. Despite the stabilizing effect, lime and cement addition did not enhance the mechanical performance as expected. 

Pure clay mortars exhibited the highest flexural and compressive strengths, outperforming their lime- and cement-stabilized variants. The addition of PETG and ABS meshes significantly increased the flexural strength of 3D-printed pure clay mortars by over 120% compared to the unreinforced 3D-printed mortars while also surpassing the molded ones by 28% and 49%, respectively. In contrast, PLA meshes resulted in a negligible increase in flexural strength due to the lower mechanical properties of PLA compared to PETG and ABS. Additionally, the PLA mesh likely exhibits weaker adhesion to the clay mortar. Mesh reinforcement proved especially effective in 5% cement mortars, with PETG and PLA meshes increasing flexural strength by over 590%.

This study underscores the importance of utilizing additive manufacturing techniques in clay mortars as a way to improve mortar production technology with reliable results. The meshes produced for this research, especially PETG and ABS, significantly enhanced the mechanical performance and cohesion of the 3D-printed clay mortars. Additionally, the presence of meshes affected the way the specimens fractured.

Future work will focus on mesh geometry and architecture, as well as on the density of the meshes in the mortar structure, testing more strategic placements within the mortar (e.g., in the upper and lower layers). Once basic parameters are understood, the research opens a new perspective on the possibilities of using new technologies in sustainable restoration by employing materials and techniques that can easily address problems with durable and compatible materials.

## Figures and Tables

**Table 1:** Characterization of binders.

	Method	Clay	Hydrated Lime	Cement
Density (g/cm^3^)	Gas pycnometry	2.609	2.279	3.105
CaO%	XRF	5.42	87.4	61.70
MgO%	XRF	2.22	0.84	1.20
SO_3_%	XRF	-	0.49	3.34
Fe_2_O_3_%	XRF	7.52	0.08	3.07
Al_2_O_3_%	XRF	15.23	0.03	3.14
SiO_2_%	XRF	58.00	-	17.54
K_2_O%	XRF	3.15	-	0.12
Na_2_O%	XRF	0.83	-	1.10
MnO%	XRF	0.13	-	-
TiO_2_%	XRF	0.73	-	-
Particle size (μm)	PSD	d (0.1): 3.261	d (0.1): 1.293	d (0.1): 2.438
		d (0.5): 30.724	d (0.5): 3.374	d (0.5): 10.248
		d (0.9): 245.651	d (0.9): 16.041	d (0.9): 28.661

**Table 2:** Content of mortar series and workability.

Preparation Method	Mortar Series	Clay	Lime	Cement	Ceramic Powder	Water	Plasticizer	Workability (cm)
Molded and 3D Printed	Lime 10%	0.9	0.1	-	0.2	0.40	2% *w*/*w* of binder	14
	Lime 20%	0.8	0.2	-	0.2	0.40		
	Cem 5%	0.95	-	0.05	0.2	0.41		
	Clay	1	-	-	0.2	0.37		

**Table 3:** Three-dimensionally printed prisms.

Mixture	Number of Prisms without Mesh	Number of Prisms with Integrated Mesh
Clay	3	3
Clay—5% Cement	3	3
Clay—10% Lime	3	-
Clay—20% Lime	3	-

## Data Availability

All the data are included in the manuscript.

## References

[1] Xu J., Ding L., Love P.E.D. (2017). Digital reproduction of historical building ornamental components: From 3D scanning to 3D printing. Autom. Constr..

[2] Jo Y.H., Hong S., Jo S.Y., Kwon Y.M. (2020). Noncontact restoration of missing parts of stone Buddha statue based on three-dimensional virtual modeling and assembly simulation. Herit. Sci..

[3] Blavier C.L.S., Huerto-Cardenas H.E., Aste N., Del Pero C., Leonforte F., Della Torre S. (2023). Adaptive measures for preserving heritage buildings in the face of climate change: A review. Build. Environ..

[4] Vidal F., Vicente R., Silva J.M. (2019). Review of environmental and air pollution impacts on built heritage: 10 questions on corrosion and soiling effects for urban intervention. J. Cult. Herit..

[5] Fabbri A., Morel J.-C. (2016). Earthen materials and constructions. Nonconventional and Vernacular Construction Materials.

[6] Yazdani Mehr S. (2019). Analysis of 19th and 20th Century Conservation Key Theories in Relation to Contemporary Adaptive Reuse of Heritage Buildings. Heritage.

[7] Turk J., Mauko Pranjić A., Hursthouse A., Turner R., Hughes J.J. (2019). Decision support criteria and the development of a decision support tool for the selection of conservation materials for the built cultural heritage. J. Cult. Herit..

[8] Armesto-González J., Riveiro-Rodríguez B., González-Aguilera D., Rivas-Brea M.T. (2010). Terrestrial laser scanning intensity data applied to damage detection for historical buildings. J. Archaeol. Sci..

[9] Moropoulou A., Labropoulos K.C., Delegou E.T., Karoglou M., Bakolas A. (2013). Non-destructive techniques as a tool for the protection of built cultural heritage. Constr. Build. Mater..

[10] Balletti C., Ballarin M. (2019). An Application of Integrated 3D Technologies for Replicas in Cultural Heritage. ISPRS Int. J. Geo-Inf..

[11] Brunetaud X., Luca L.D., Janvier-Badosa S., Beck K., Al-Mukhtar M. (2012). Application of digital techniques in monument preservation. Eur. J. Environ. Civ. Eng..

[12] Bayram B., Nemli G., Özkan T., Oflaz O.E., Kankotan B., Çetin İ. (2015). Comparison Of Laser Scanning And Photogrammetry and Their Use For Digital Recording Of Cultural Monument Case Study: Byzantine Land Walls-Istanbul. ISPRS Ann. Photogramm. Remote Sens. Spat. Inf. Sci..

[13] Costamagna E., Quintero M.S., Bianchini N., Mendes N., Lourenço P.B., Su S., Paik Y.M., Min A. (2020). Advanced non-destructive techniques for the diagnosis of historic buildings: The Loka-Hteik-Pan temple in Bagan. J. Cult. Herit..

[14] Kęsik J., Milosz M., Montusiewicz J., Samarov K. (2021). Documenting the geometry of large architectural monuments using 3D scanning—The case of the dome of the Golden Mosque of the Tillya-Kori Madrasah in Samarkand. Digit. Appl. Archaeol. Cult. Herit..

[15] Laing R., Leon M., Isaacs J. Monuments Visualization: From 3D Scanned Data to a Holistic approach, an Application to the City of Aberdeen. Proceedings of the 2015 19th International Conference on Information Visualisation.

[16] Higueras M., Calero A.I., Collado-Montero F.J. (2021). Digital 3D modeling using photogrammetry and 3D printing applied to the restoration of a Hispano-Roman architectural ornament. Digit. Appl. Archaeol. Cult. Herit..

[17] Balletti C., Ballarin M., Guerra F. (2017). 3D printing: State of the art and future perspectives. J. Cult. Herit..

[18] Kantaros A., Ganetsos T., Petrescu F.I.T. (2023). Three-Dimensional Printing and 3D Scanning: Emerging Technologies Exhibiting High Potential in the Field of Cultural Heritage. Appl. Sci..

[19] Sargentis G.-F., Frangedaki E., Chiotinis M., Koutsoyiannis D., Camarinopoulos S., Camarinopoulos A., Lagaros N.D. (2022). 3D Scanning/Printing: A Technological Stride in Sculpture. Technologies.

[20] Vailati M., Mercuri M., Angiolilli M., Gregori A. (2021). Natural-Fibrous Lime-Based Mortar for the Rapid Retrofitting of Heritage Masonry Buildings. Fibers.

[21] Vailati M., Mercuri M., Gregori A. (2024). Out-of-plane non-intrusive seismic retrofitting of in-plane damaged masonry infill through 3D printed recycled plastic devices. Constr. Build. Mater..

[22] Vailati M., Gregori A., Mercuri M., Monti G. (2023). A non-intrusive seismic retrofitting technique for masonry infills based on bed-joint sliding. J. Build. Eng..

[23] Sustainable Robots 4D Printing-Soleimanzadeh-2023-Advanced Sustainable Systems-Wiley Online Library. https://onlinelibrary.wiley.com/doi/full/10.1002/adsu.202300289.

[24] Bezzi F., Fabbri P., Magnani G., Salernitano E., Scafè M., Strafella A. (2022). Aqueous aluminium titanate paste for the liquid deposition modelling. Open Ceram..

[25] Goh G.D., Yap Y.L., Agarwala S., Yeong W.Y. (2019). Recent Progress in Additive Manufacturing of Fiber Reinforced Polymer Composite. Adv. Mater. Technol..

[26] Feilden B. (2003). Conservation of Historic Buildings.

[27] Tsyrfa I., Serbina N., Meteliev I., Goussous J., Chung J.-K. (2024). Issues of preservation and restoration of historical monuments in the occupied territories. Int. J. Environ. Stud..

[28] Šekularac N., Debljović Ristić N., Mijović D., Cvetković V., Barišić S., Ivanović-Šekularac J. (2019). The Use of Natural Stone as an Authentic Building Material for the Restoration of Historic Buildings in Order to Test Sustainable Refurbishment: Case Study. Sustainability.

[29] Song R., Wang Y., Tang Y., Peng J., Liu J., Yang C. (2022). 3D Printing of natural sandstone at pore scale and comparative analysis on micro-structure and single/two-phase flow properties. Energy.

[30] Feng X.-T., Gong Y.-H., Zhou Y.-Y., Li Z.-W., Liu X.-F. (2019). The 3D-Printing Technology of Geological Models Using Rock-Like Materials. Rock Mech. Rock Eng..

[31] Sharafisafa M., Shen L., Xu Q. (2018). Characterisation of mechanical behaviour of 3D printed rock-like material with digital image correlation. Int. J. Rock Mech. Min. Sci..

[32] Paul S.C., van Zijl G.P.A.G., Tan M.J., Gibson I. (2018). A review of 3D concrete printing systems and materials properties: Current status and future research prospects. Rapid Prototyp. J..

[33] Moretti M., Rangel B., Guimarães A.S., Lino J., Santana L. (2023). WASP in the Edge of 3D Printing. 3D Printing for Construction with Alternative Materials.

[34] Chen Y., He S., Zhang Y., Wan Z., Çopuroğlu O., Schlangen E. (2021). 3D printing of calcined clay-limestone-based cementitious materials. Cem. Concr. Res..

[35] Freire T., Brun F., Mateus A., Gaspar F., Rodrigues H., Gaspar F., Fernandes P., Mateus A. (2021). 3D Printing Technology in the Construction Industry. Sustainability and Automation in Smart Constructions.

[36] Chen Y., Figueiredo S.C., Li Z., Chang Z., Jansen K., Çopuroğlu O., Schlangen E. (2020). Improving printability of limestone-calcined clay-based cementitious materials by using viscosity-modifying admixture. Cem. Concr. Res..

[37] Manikandan K., Wi K., Zhang X., Wang K., Qin H. (2020). Characterizing cement mixtures for concrete 3D printing. Manuf. Lett..

[38] Zhang X., Li M., Lim J.H., Weng Y., Tay Y.W.D., Pham H., Pham Q.-C. (2018). Large-scale 3D printing by a team of mobile robots. Autom. Constr..

[39] Puzatova A., Shakor P., Laghi V., Dmitrieva M. (2022). Large-Scale 3D Printing for Construction Application by Means of Robotic Arm and Gantry 3D Printer: A Review. Buildings.

[40] Alabbasi M., Agkathidis A., Chen H. (2023). Robotic 3D printing of concrete building components for residential buildings in Saudi Arabia. Autom. Constr..

[41] Xu W., Huang S., Han D., Zhang Z., Gao Y., Feng P., Zhang D. (2022). Toward automated construction: The design-to-printing workflow for a robotic in-situ 3D printed house. Case Stud. Constr. Mater..

[42] Cao X., Yu S., Cui H., Li Z. (2022). 3D Printing Devices and Reinforcing Techniques for Extruded Cement-Based Materials: A Review. Buildings.

[43] Gomes M.I., Faria P., Gonçalves T.D. (2018). Earth-based mortars for repair and protection of rammed earth walls. Stabilization with mineral binders and fibers. J. Clean. Prod..

[44] Papayianni I., Pachta V., Stefanidou M. (2013). Analysis of ancient mortars and design of compatible repair mortars: The case study of Odeion of the archaeological site of Dion. Constr. Build. Mater..

[45] Papayianni I., Mortars for Intervention in Monuments and Historical Buildings (2003). WIT Transactions on the Built Environment, Jan. https://www.academia.edu/100434523/Mortars_For_Intervention_In_Monuments_And_Historical_Buildings.

[46] (2019). Methods of Test for Mortar for Masonry—Part 11: Determination of Flexural and Compressive Strength of Hardened Mortar.

[47] (2007). Ente Italiano di Normazione, Metodi di Prova per Malte per Opere Murarie-Parte 3: Determinazione della Consistenza della Malta Fresca (Mediante Tavola a Scosse).

[48] Lu C., Qi M., Islam S., Chen P., Gao S., Xu Y., Yang X. (2018). Mechanical performance of 3D-printing plastic honeycomb sandwich structure. Int. J. Precis. Eng. Manuf. Green. Tech..

[49] Baz B., Aouad G., Khalil N., Remond S. (2021). Inter-layer reinforcement of 3D printed concrete elements. Asian J. Civ. Eng..

[50] Geneidy O., Kumarji S., Dubor A., Sollazzo A., Bos F.P., Lucas S.S., Wolfs R.J.M., Salet T.A.M. (2020). Simultaneous Reinforcement of Concrete While 3D Printing. Second RILEM International Conference on Concrete and Digital Fabrication.

[51] Karozou A., Pavlidou E., Stefanidou M. (2019). Enhancing Properties of Clay Mortars Using Nano-Additives. Solid State Phenom..

[52] Gomes M.I., Gonçalves T.D., Faria P., Mileto C., Vegas F., Cristini V. (2012). Earth-based repair mortars: Experimental analysis with different binders and natural fibers: 1st International Conference on Rammed Earth Conservation (RESTAPIA). Rammed Earth Conservation.

[53] (2021). Testing Concrete in Structures—Part 4: Determination of Ultrasonic Pulse Velocity.

[54] Begarin F., Garrault S., Nonat A., Nicoleau L. (2011). Hydration of alite containing aluminium. Adv. Appl. Ceram..

[55] Barret P., Bertrandie D. (1986). Fundamental hydration kinetic features of the major cement constituents: Ca_3_SiO_5_ and βCa_2_SiO_4_. J. Chim. Phys..

[56] Manzano H., Dolado J.S., Guerrero A., Ayuela A. (2007). Mechanical properties of crystalline calcium-silicate-hydrates: Comparison with cementitious C-S-H gels. Phys. Status Solidi A.

